# Suitability and Profitability of a Cereal Aphid for the Parasitoid *Aphidius platensis* in the Context of Conservation Biological Control of *Myzus persicae* in Orchards

**DOI:** 10.3390/insects11060381

**Published:** 2020-06-19

**Authors:** Jeniffer K. Alvarez-Baca, Armando Alfaro-Tapia, Blas Lavandero, Cécile Le Lann, Joan Van Baaren

**Affiliations:** 1 Laboratorio de Control Biológico, Instituto de Ciencias Biológicas, Universidad de Talca, Talca 3460000, Chile; jealvarez@utalca.cl (J.K.A.-B.); aalfaro@utalca.cl (A.A.-T.); 2 UMR 6553 Ecobio, Centre National de la Recherche Scientifique, Université de Rennes 1, 263 Avenue du Général Leclerc, 35042 Rennes, France; cecile.lelann@univ-rennes1.fr (C.L.L.); joan.van-baaren@univ-rennes1.fr (J.V.B.)

**Keywords:** biological control, parasitoids, host selection, profitability, fitness

## Abstract

The use of cover crops can promote the abundance and early arrival of populations of natural enemies. Cereal cover crops between orchards rows could encourage the early arrival of the parasitoid *Aphidius platensis*, as they offer alternative winter hosts (e.g., *Rhopalosiphum padi*), enhancing the control of *Myzus persicae* in spring. However, the preference for and suitability of the alternative host must be addressed beforehand. To evaluate the potential of this strategy, we assessed host preference using behavioural choice tests, as well as no-choice tests measuring fitness traits, when developing on both host species. One source field for each aphid population from the above hosts was chosen. There was a clear choice for *R. padi* compared to *M persicae*, independently of the source, probably due to more defensive behaviours of *M. persicae* (i.e., kicks and escapes). Nevertheless, both aphid species were suitable for parasitoids’ development. The female progeny developed on *R. padi* were larger in size, irrespective of their origin. According to our results, in peach orchards with cereals sown between peach trees during the autumn, where we expect when *R. padi* populations will no longer be available during spring, *A. platensis* should be able to switch to *M. persicae*.

## 1. Introduction

Cover crops can be used as a habitat management strategy to enhance natural enemies’ populations of a target pest, favoring natural enemies by means of different mechanisms. The mechanisms behind this proposed strategy, include providing shelter, from deleterious environmental factors such as pesticides, harvest, extreme weather and cultivation, among others, as well as providing host (other suitable species) and non-host resources such as nectar, pollen and honeydew [1,2,3,4,5,6].The use of this strategy has been increasing in recent years, in Europe [7,8] America [9,10], Asia and Africa [11,12,13,14,15] and Latin America [16]. Additionally, cover crops can provide conditions for the temporal synchronization of natural enemies and the target pest by means of the aforementioned mechanism, increasing natural enemy populations before pest arrival on the target crop [17,18,19,20]. This habitat management strategy has been shown to increase crop yields [6,20,21] use of a mixture of cereal cover crops (*Festuca arundinacea* (Schreb)*, Poa* sp.(Linnaeus)*, Bromus* sp.(Linnaeus)*,* etc.) in citrus orchards in Spain has been used to control the main aphid pest, *Aphis spiraecola* (Patch) (Hemiptera: Aphididae), and has been shown to significantly advance the arrival of predators [22] Similarly, in a study in New Zealand, the use of cover plants like alyssum (*Lobularia maritima* (Linnaeus)), buckwheat (*Fagopyrum esculentum* (Moench)) and phacelia* (Phacelia tanacetifolia* (Benth)) in apple orchards, increased the parasitism rates of the larvae of the light-brown apple moth, *Epiphyas postvittana* (Walker) (Lepidoptera: Tortricidae) in alyssum and buckwheat treatments compared to controls [23]. Prior to their establishment, an important aspect of cover crops to take into account is that they do not enhance the target pest [22]. Alternative hosts/prey must impact life history traits and development of the natural enemy species positively, but not affect their oviposition/feeding preference in relation to the target pest species [24]. Therefore, in order to correctly implement such a strategy, the alternative hosts/prey for the target natural enemies must be viable from a preference and a performance perspective.

Regarding host quality, different theories can explain the choices of a female parasitoid for oviposition. The *optimal foraging theory* predicts that a foraging female parasitoid will always prefer the most profitable host [25]. This is in relation to the *preference-performance hypothesis* (PPH), also known as the “mother knows best” hypothesis [26], which states that host preference in female parasitoids is positively correlated to the developmental success of its progeny [27,28,29,30,31]. However, in some cases, female inexperience or defensive behaviours of the host which can decrease its profitability by increasing the handling time [32] for example, it can lead to choices that can seem suboptimal [33]. It is also known that specialist parasitoids are more efficient at exploiting a host than generalist ones [34]. Parasitoids which are particularly locally adapted, can exhibit a higher performance and preference in relation to their target host, being more efficient [35,36]. The lower efficiency of generalist parasitoids can be avoided by host fidelity, which is traduced into preferentially attacking the same host species as those from which they emerged (i.e., natal hosts) [37]. Host fidelity allows a quicker recognition of the host, maximizing the reproductive success and survival of the progeny [38,39]. As the host fidelity of female parasitoids could result in more parasitoids remaining on their natal host and less host shift to alternative hosts [40], it is extremely important to be aware of this information in advance.

In Chile, 169 aphid species have been reported, with more than 100 of them having been introduced [41,42]. Most of these species constitute important agricultural pests in cereals, legumes and fruit orchards [43,44,45,46]. An economically important agricultural pest is the peach-potato aphid, *Myzus persicae* (Sulzer) ( Hemiptera, Aphididae)*,* which frequently exhibits resistance to insecticides on several crops, including their primary host, the peach, *Prunus persica* (Linnaeus) [43,47,48]. On the other hand, *Rhopalosiphum padi* (Linnaeus) (Hemiptera, Aphididae) is an important insect pest attacking several host plants of wild and graminaceous crops, including wheat, oat and barley [47]. The polyphagous endoparasitoid *Aphidius platensis* Brethes (Hymenoptera, Braconidae) attacks several aphid species, and it is known to parasitize aphids that feed on different plant species, namely cultivated grasses, vegetables and fruit trees, including hosts of economic importance such as *M. persicae* and *R. padi* [49,50]. A recent survey in cereal fields during winter showed that *R. padi*’s most abundant parasitoid is *A. platensis*, which represents 70% of the parasitoids emerging from the mummies (Alfaro-Tapia et al. unpublished data). It has been suggested that the populations of *R. padi* abandon cereal crops in early spring [51], forcing *A. platensis* to search for other host species. On the other hand, *M. persicae* populations increase on several *Prunus* species in early spring, and *A. platensis* has been collected and observed parasitizing *M. persicae* and *Brachycaudus helichrysi* Kaltenbac and *Aphis spiraecola* Patch (Hemiptera, Aphididae) on *Prunus spp.* orchards (Alvarez-Baca et al. unpublished).

In order to verify whether the parasitoid *A. platensis* effectively has the ability to switch from an alternative winter host *R. padi* to a spring target host *M. persicae*, we studied the preference and performance of *A. platensis* on the two aphid-plant complex species in the laboratory. We hypothesized that: (i) in a choice situation, the origin of the host of the parasitoid will not influence its choice: e.g., *A. platensis* females will show no host fidelity, as they are adaptive in order to be able to change hosts according to their relative availability during the season (ii) in a non-choice situation, both aphids will be suitable hosts in terms of survival and development for *A. platensis,* as parasitoid populations were collected and reared on both hosts. Therefore,* M. persicae* can be accepted by the female parasitoids independently of their origin.

## 2. Materials and Methods

### 2.1. Insect Rearing

#### 2.1.1. Parasitoids

*Aphidius platensis* is a species belonging to the *Aphidius colemani* group [52]. It was initially believed to originate from South America (classified as a member of the Neotropical Faunistic Complex) [53], however, a few years later, it was documented as being of Indian origin [50], with a consequent expansion to other continents, currently being present in North Africa, Australia, Middle East and South America [54]. In this present study, two *A. platensis* populations were established for laboratory rearing. One parasitoid population came from *R. padi* mummies (i.e., parasitized aphids), collected during the winter season (July–August) on winter cereals (Talca, 35°35′28.66″ S, 71°28′26.5″ W and 189 m). The other population came from *M. persicae* mummies, collected at the beginning of the spring season (September–October) from peach orchards (Duao 35°34′23.23″ S and 71°33′09.53″ W 183 m). Parasitoids were determined using taxonomic keys [49,52] and once the population was established, a molecular approach with partial sequences of mtCOI [52,55] was carried out on the progeny to confirm the determination of the species. Parasitoids were fed with 30% honey solution through soaked cotton wicks and water. Parasitoids were maintained on their respective hosts until the experiments were carried out. New aphids, honey and water were added on a weekly basis to ensure a constant supply of aphids to parasitoids. Rearing and all experiments were maintained under controlled conditions (20 ± 1 °C, 65 ± 10% RH and 16:8 h day/night).

#### 2.1.2. Aphid Hosts

A polyclonal mass rearing of *R. padi* on potted wheat *Triticum aestivum* (Linnaeus) v. Saturnus and *M. persicae* on mustard *Sinapis alba* (Linnaeus) were established in the ECOBIO Laboratory in separated Plexiglas cages of 40 cm^3^. Aphids of both species were used as host resources for parasitoids, as well as in the experiments. Plants and aphids were maintained under summer and controlled laboratory conditions (20 ± 1 °C, 65 ± 10% RH and 16:8 h day/night).

#### 2.1.3. Insect Material for the Experiments

To standardize parasitoid females used in the experiments, *A. platensis* mummies were isolated in small plastic tubes (2 mL) until emergence. Parasitoid emergence was checked once daily at the same time. After emergence, adult parasitoids were sexed and females were left with one to two males for mating over a 24-hour period in micro-cages (h = 20 cm, Ø = 5 cm). They were fed with 30% honey-water solution using a cotton wick. Virgin females with less than 24 h old were used in the experiments.

A preliminary experiment was performed to standardize the size of the aphids used in the experiments, therefore, 20 aphids of second and third instars were measured for each of the two-aphid host species (total *n* = 80 aphids). The fresh body mass of each individual was measured with a microbalance (XP2U, ± 0.0001 mg, Mettler Toledo, Columbus, OH, USA). After weighing, each aphid hind tibia length was measured with the numeric image analysis software ImageJ (National Institutes of Health, Bethesda, MD, USA), under a binocular system (SMZ800, Nikon, Tokyo, Japan. with 65× of total magnification) linked to a camera video (JVC KY-F50, JVC Pro, Paris, France). The difference among aphid instars was analyzed by using a Wilcoxon signed rank test.

### 2.2. Host Preference Assay: Choice Experiment

The host preference of *A. platensis* was studied by placing one second instar aphid of *R. padi* and one third instar of *M. persicae* in a Petri dish arena (Ø = 2.5 cm), each on a fresh and non-previously infested leaf of their host plant. The size was chosen because it is in the range used in several choice experiments (between 2–4 cm) as in [51,56]. Besides, this arena size fitted perfectly to the focus distance and field view of our lens, facilitating the complete observation of the behaviours recorded without moving the arena. Each aphid was placed on a plantlet of 10 cm high at least 30 min before the experiments started, in order to allow its establishment on the plant, as in [57]. A piece of the leave with the aphid was cut 5 min before the experiment and placed in the observation arena. Female parasitoids were introduced into the arena after aphid establishment. The experiment began once the female was released, and ended when the female made a choice (host acceptance), following [58]. During the experiment, the following parasitoid behaviours were used: (i) *“first aphid perceived*” which can be followed by either rejection (changing direction without contact, before reaching the aphid) or continuation to the next behaviour, (ii) *“antennal evaluation”*, when the parasitoid is moving its antennae just above the aphid or touching the aphid with at least one of its antennae, which is followed by rejection or continuation to the next behaviour, (iii) *“abdomen preparation”*, when the female curved its abdomen in a forward position and could touch the host with its abdomen without any insertion [51,56], (iv) *“ovipositor probing”* (insertion of the ovipositor not resulting in a successful oviposition); this step can be facultative; (v) “*wing fluttering*”, showing a continuous movement of the wings and (vi) *“host acceptance”* (ovipositor insertion with abdomen bending and antennae backward, i.e., oviposition). *Host handling time for oviposition* was considered as the mean time from the encounter (which is followed by antennal evaluation) to a successful oviposition [56]. Additionally, aphids also showed a variety of defensive behaviours in response to the parasitoid´s attack that could also lead to rejection from the female [59]. Among aphid defensive behaviours, *kicking* with the legs, *escaping* (walking away) and *cornicle secretion* (at the end of the experiment) were registered. All the behaviours were recorded with the “Etholog” package (v2.2) [60]. After each female choice, both aphid hosts and plant material were replaced, and the experiment was repeated until five different aphids were parasitized by the same female to avoid any hazardous and unpredictable decisions made by the female. The positions of the aphid host species were exchanged at each choice test. A total of 15 females emerging from *R. padi* and 15 from *M. persicae* were used during the experiments. If after 15 min the female remained inactive, it was replaced by a new female and discarded from the analyses (which happened only once). Within one hour of the experiments, the aphids attacked by females (ovipositor insertion) were dissected to check for parasitoid eggs, in order to ensure that the attack had resulted in a successful oviposition, as in [58].

### 2.3. Profitability Assay: Non-choice Experiment

In order to maximize their fitness, females look for the most profitable host, therefore, we use the term “profitability” as an equivalent of the fitness in the progeny as given in [61]. The aim of this experiment was to measure the performance of parasitoids on both hosts according to their original host. The experiment was carried out on two populations of aphids (*R. padi* and *M. persicae*) and two populations of parasitoids (from *R. padi* and from *M. persicae*). Micro-cages (h = 20 cm, Ø =5 cm) containing 10 cm high wheat or 10 cm mustard plantlets with at least two leaves, were infested with 30 s instar aphids of *R. padi* or 30 third instar aphids of *M. persicae*, respectively. Once the aphids were established on the plants, a female was released into each micro-cage and left to parasitize them for 24 h. After this time, the female was removed and the aphids were monitored until their mummification. Ten replicates per host aphid and per origin of the parasitoid were performed (*n* = 40 females) over two consecutive weeks (five replicates of all the combinations per week). The micro-cages containing the potentially parasitized aphids were checked daily, and once the mummies were formed, they were isolated individually into gelatin capsules (Ø = 7 mm, l = 1.8 cm; Capsugel Coni-snap 1EL, Morristown, NJ, USA) until parasitoid emergence. Different parameters were evaluated: the *parasitism rate* was calculated by dividing the number of mummies formed by the total number of aphids offered to each female [62]; the *emergence rate* was calculated as the proportion of parasitoids which emerged from the mummies formed [63]; the *developmental time* was estimated as the time from oviposition to the emergence of an adult parasitoid, divided between the development time from oviposition (egg) to mummification (larval development) and time from mummification to emergence (nymphal development) as in [58]. Finally, the *fresh body mass* and *tibial lengths* of all the emergent individuals were measured. When measuring fresh body mass and tibial lengths, we followed the aforementioned procedure for the aphids. These traits of all emerged progeny were measured during each treatment.

### 2.4. Statistical Analysis

#### 2.4.1. Choice Experiment

As each female was tested in five aphid choice tests, generalized estimating equations (i.e., GEE) were used [64] as they allow correlations between repeated measures of a dependent variable to be taken into account [65]. For the analysis, the behavioural parameters were divided into two modalities: The first analysis grouped the First aphid perceived and Host acceptance, which aimed to test whether a female parasitoid from one origin (*R. padi* or *M. persicae*) chooses its original host or the alternative, according to the origin of the female (fixed factor). The second analysis grouped the remaining behaviours (e.g., Ovipositor probing, Wing fluttering, etc.), and in addition to the origin of the female evaluated in the first analysis, we considered whether this origin makes the female perform these behaviours differently when facing one or the other host (female origin host and the aphid tested as fixed factors). The interaction between these two explanatory variables on each dependent variable (behaviours) was also considered in all cases. *Exchangeable* correlation working matrices were used, as no specific patterns for the presentation of the five pairs of aphids was assumed for the GEEs [58]. To select this correlation, we checked previously that the sequence of oviposition choices for each female did not have any influence on the parasitoid behavioural choices. The first aphids perceived and accepted (host acceptance) by the parasitoids were analyzed as the proportion of perceptions/successful ovipositions respectively, with a binomial error and a logit-link function for proportional data [66].The mean time of wing fluttering and handling time for oviposition were analyzed assuming a Gaussian error and an identity link function. Wing fluttering was analyzed as the mean time the female spent fluttering, considering all the encounters, including those which did not end in a successful oviposition. The presence/absence of cornicular secretions at the end of each assay was analyzed assuming a binomial error and a logit-link function for proportional data [66]. In addition, the number of ovipositor probing (number of stings), aphid kicking (number of aphid kicks) and aphid escaping (number of escapes) were compared using a Poisson error and a log-link function for count data.

#### 2.4.2. Profitability Assay

In this assay, we evaluated parasitoid development and fitness proxy variables; the origin of the female and the aphid species chosen were considered as fixed factors, as was the interaction between these two explanatory variables, and we considered the identity of the female as a random factor. The percentage of emergence was compared using a GLMM, assuming a binomial error and a logit-link function. The total developmental time was analyzed with a binomial error and a logit-link function [67]. The parasitism rate was analyzed with a quasibinomial (GLM) error and a logit-link function for proportional data (for overdispersed data). Fresh body mass and the tibial length of the progeny were analyzed using a GLMM, assuming a Gaussian error and an identity link function. In all the cases, models with interactions between the fixed factors were compared using the Akaike information criterion (AIC), and if insignificant, they were not included in the model.

All statistical analyses were performed with the R v.3.5.1 software (R Development Core Team, Vienna, Austria, 2018). For the choice experiment, we used the *geepack* package for the GEE function. For the profitability assay, we used the *lme4* package for mixed models [68] and the *car* package for differences between models with the AIC criterion. To establish significant differences, we used a ‘Tukey’ test, correcting for multiple comparisons by the ‘single-step’ method, using the *Multcomp* package [69].

## 3. Results

### 3.1. Preliminary Experiment

*R. padi* second instars had a fresh body mass of 0.055 ± 0.002 mg and a tibia length of 0.336 ± 0.008 mm, whereas third instars had a fresh body mass of 0.124 ± 0.005 mg and a tibia length of 0.519 ± 0.010 mm. In the case of *M. persicae*, second instars had a fresh body mass of 0.049 ± 0.001 mg and a tibia length of 0.359 ± 0.006 mm; third instars on the other hand presented a fresh body mass of 0.056 ± 0.001 mg and a tibia length of 0.407 ± 0.009 mm. Second and third instars of *R. padi* were heavier than the respective instars of *M. persicae* (second instars: W = 120, *p* = 0.03; third instars: W = 0, *p* < 0.01). However, as the second instars of *R. padi* had a similar fresh body mass to the third instars of *M. persicae* (Wilcoxon matched paired test, W = 157, *p* = 0.249), these were finally selected for this study.

### 3.2. Choice Experiment

**Figure 1:**
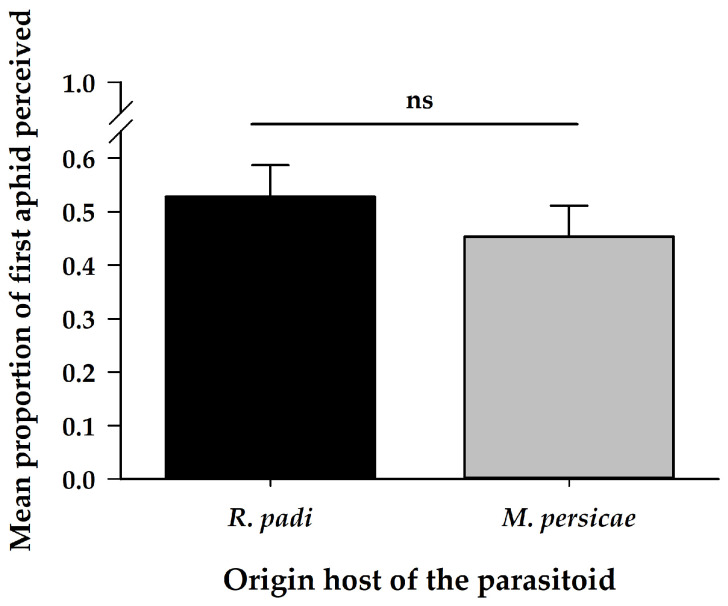
Mean proportion (±SE) of first aphid perceptions by *A. platensis* from two origins (*R. padi* and *M. persicae)* exposed to these aphid hosts in a paired arena. Asterisks indicate significant differences: ‘ns’ non-significant *p* > 0.05.

Female parasitoid first aphid perception was similar in relation to both aphid species regardless of their origin host (Table 1) (Figure 1). 

**Figure 2:**
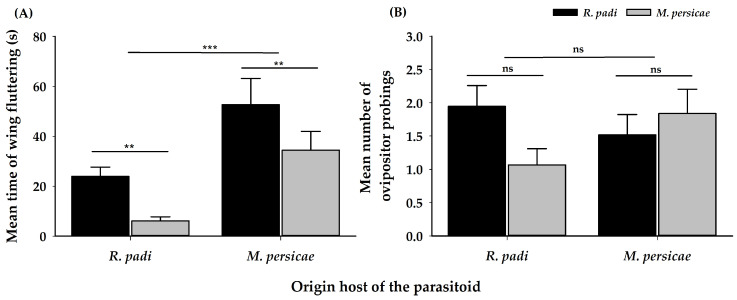
(**A**) Mean time wing fluttering (±SE) in seconds, and (**B**), mean number of ovipositor probings (±SE) of *A. platensis* from two origins (*R. padi* and *M. persicae*) exposed to these aphid hosts in a paired arena. Asterisks indicate significant differences: ‘***’ *p* < 0.001, ‘**’ *p* < 0.01, ‘ns’ non-significant *p* > 0.05. Black and grey bars show the chosen/tested host.

Immediately after antennal contact, parasitoid females spent more time wing fluttering when attacking *R. padi*, than when attacking *M. persicae*. More time was spent fluttering overall in the case of females of *M. persicae* origin (Table 1) (Figure 2A). 

**Figure 3:**
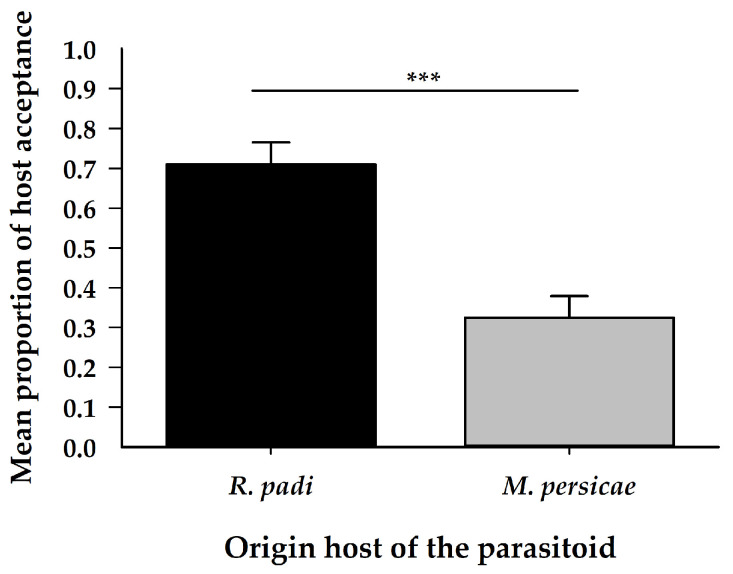
Mean proportion (±SE) of chosen aphids according to the origin of *A. platensis* females. Asterisks indicate significant differences: ‘***’ *p* < 0.001.

**Figure 4:**
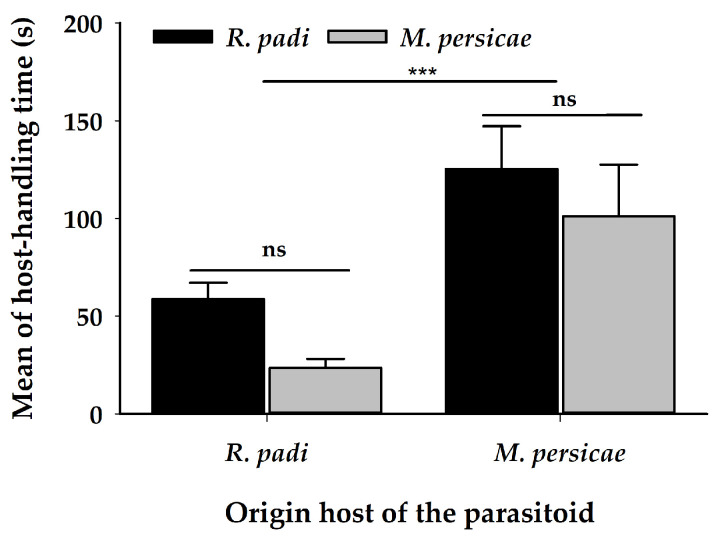
Mean of handling time for oviposition (±SE) in seconds of *A. platensis* from two origins (*R. padi* and *M. persicae*) exposed to these aphid hosts in a paired arena. Asterisks indicate significant differences: ‘***’ *p* < 0.001, ‘ns’ non-significant *p* > 0.05. Black and grey bars show the chosen/tested host.

There was no difference in the number of sting attempts (ovipositor probings) (Table 1) (Figure 2B), females attacked both aphid species, irrespective of their origin host. The proportion of successful ovipositions (host acceptance) was greater on *R. padi* than on *M. persicae*, regardless of the origin of the female parasitoid (Table 1) (Figure 3). The handling time for oviposition was different, according to the origin of the female parasitoid; females coming from *R. padi* took less time handling a host before an oviposition on both tested hosts was successful, by comparison with females originating from *M. persicae* (Table 1) (Figure 4).

**Figure 5:**
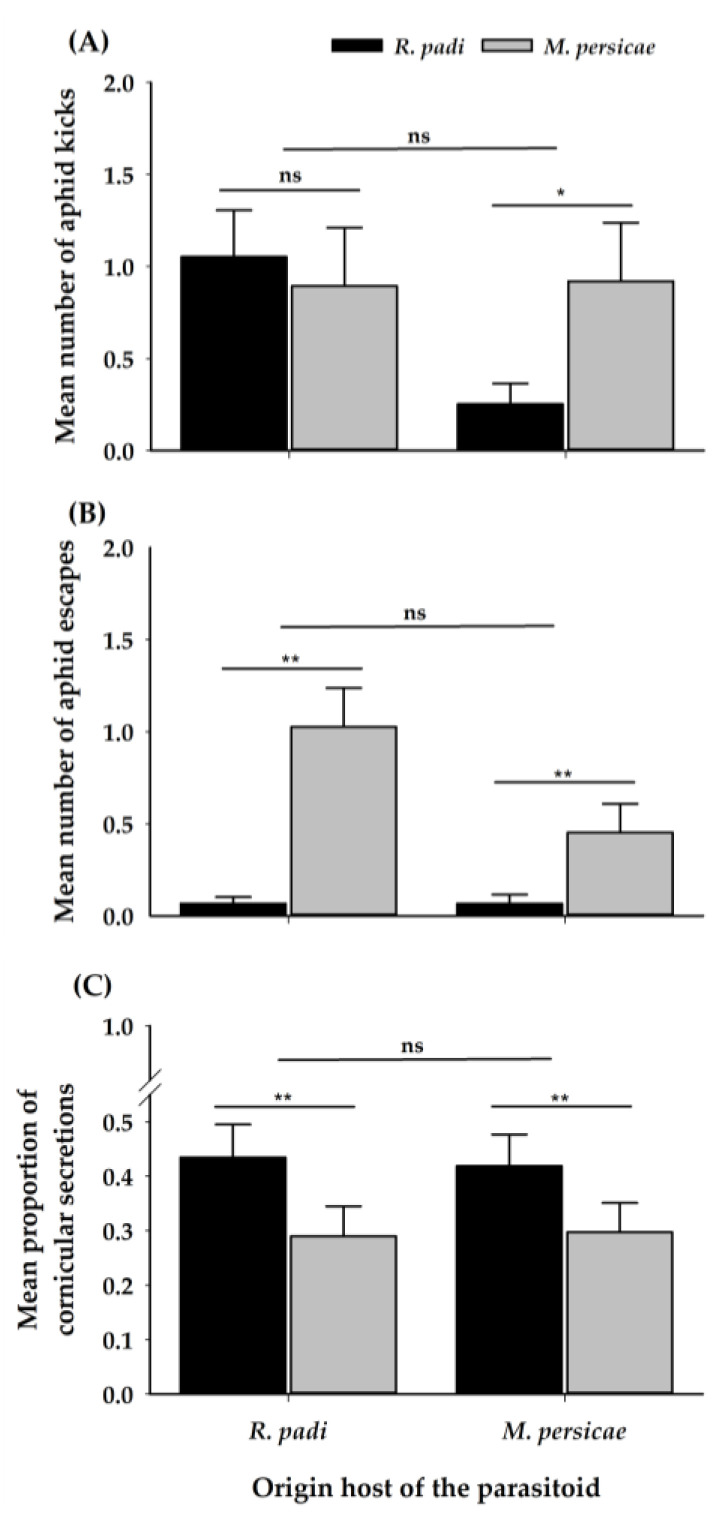
Mean (±SE) of aphid defensive behaviours: (**A**) Number of aphid kicks; (**B**) Number of aphid escapes and (**C**) Proportion of an encounter with cornicular secretions of *R. padi* and *M. persicae* when attacked by *A. platensis* coming from the two origins. Asterisks indicate significant differences: ‘**’ *p* < 0.01, ‘*’ *p* < 0.05, ‘ns’ non-significant *p* > 0.05. Black and grey bars show the chosen/tested host.

The parasitoid attack of aphids frequently resulted in aphid defensive behaviours; approximately 56% of *R. padi* and 55% of *M. persicae* responded to at least one of the three defensive behaviours evaluated. However, *M. persicae* was more defensive than *R. padi* when facing the female parasitoid, irrespective of the female origin. Therefore, *R. padi* kicked less than *M. persicae* when attacked by females coming from *M. persicae* (Table 1) (Figure 5A). Likewise, *M. persicae* escaped more times when attacked (Table 1) (Figure 5B). Despite the origin of the female, *R. padi* produced more cornicular secretions when attacked by the females (Figure 5C).

### 3.3. Profitability Assay

**Figure 6:**
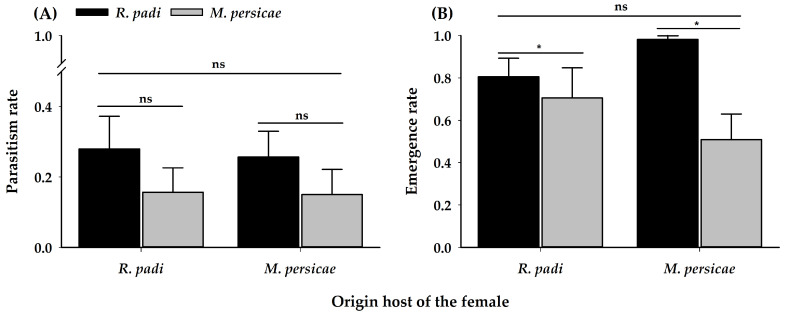
(**A**) Parasitism rate (mean ± SE) (i.e., proportion of aphid mummies formed) and (**B**) Emergence rate (mean ± SE) of adult progeny of *A. platensis* from two origins parasitizing the same aphid host. Asterisks indicate significant differences: ‘*’ *p* < 0.05, ‘ns’ non-significant *p* > 0.05. Black and grey bars show the chosen/tested host.

**Figure 7:**
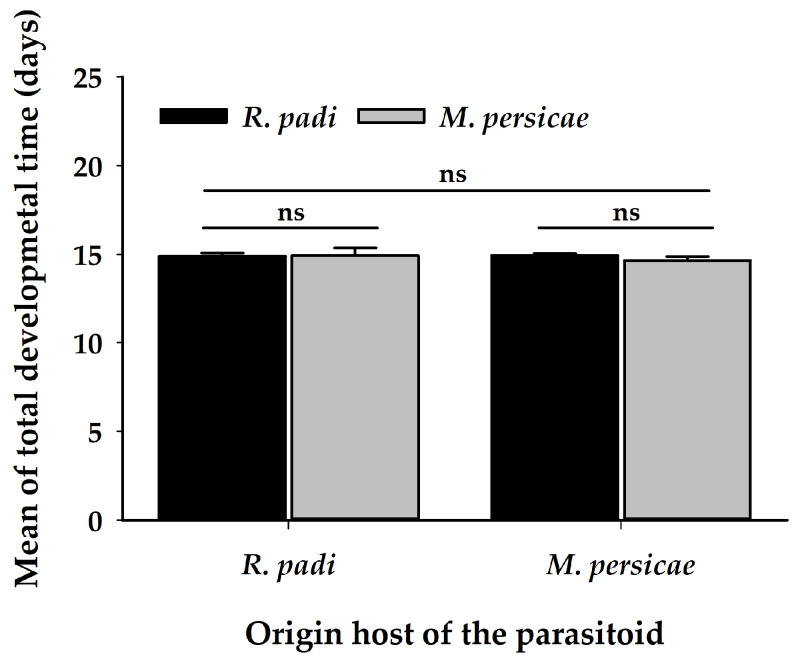
Total developmental time (mean ± SE) of *A. platensis* emerged from *R. padi* and *M. persicae* and coming from two origins (*R. padi* and *M. persicae*). Asterisks indicate significant differences: ‘ns’ non-significant *p* > 0.05. Black and grey bars show the chosen/tested host.

No significant differences were observed for the parasitism rate (Table 2) (Figure 6A). Females of *A. platensis* parasitized at a similar rate in both aphid species, regardless of their origin host. Otherwise, differences were observed in the proportion of emerged adults, with a greater proportion of progeny of emerged parasitoids when developing on *R. padi*, irrespective of the origin of the parasitoid mother (Table 2) (Figure 6B). In addition, there were no differences in the total developmental time of the parasitoid progeny (Table 2) (Figure 7) regardless of the origin of the female.

**Figure 8:**
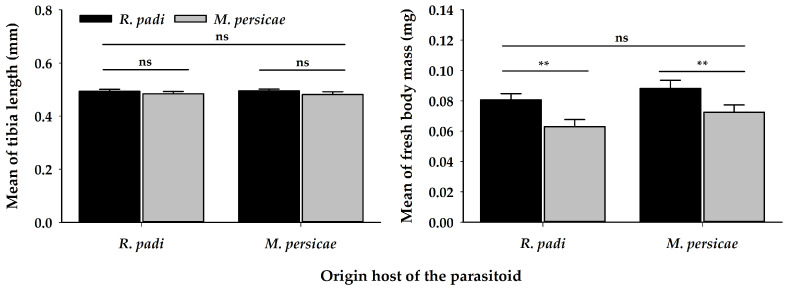
Mean of (**A**) Tibia length and (**B**) Fresh body mass (±SE) of *A. platensis* emerged from *R. padi* and *M. persicae* and coming from two origins. Asterisks indicate significant differences: ‘**’ *p* < 0.01, ‘ns’ non-significant *p* > 0.05. Black and grey bars show the chosen/tested host.

Additionally, fitness traits were evaluated on the emerging progeny in all treatments. There was no difference in the tibia length of the progeny (Table 2) (Figure 8A), irrespective of the origin host of the mother. However, regardless of the origin of the female, the progeny developing on *R. padi* was heavier than those developing on *M. persicae*. (Table 2) (Figure 8B).

## 4. Discussion

We evaluated the preference and performance of *A. platensis* individuals, originating from two aphid host populations: *R. padi* from cereals and *M. persicae* from *P. persica.* Our first hypothesis was that parasitoid females will not exhibit host fidelity. Regardless of their origin host, parasitoids preferred *R. padi,* and this seems to be due to the fact that this aphid species was less defensive. Therefore, we do not see an effect of the origin of the female parasitoids (from *R. padi* or from *M*. *persicae*). Our results in non-choice experiments, as expected, confirmed our second hypothesis, as females of both origins were physiologically able to use *M. persicae* as a host, however, parasitoid progeny emergence was higher and emerging individuals were heavier when developed on *R. padi,* an important feature in parasitoid fitness [31,70,71,72]. The implications of this preference for *R. padi,* regardless of the host origin and the suitability of both aphid species for the development of the biological control of *M. persicae* in the field, are discussed further.

Contrary to host fidelity predictions, *A. platensis* has a clear preference for *R. padi,* regardless of the origin host. This lack of host fidelity was found in other studies [73,74] of the *A. colemani* complex, reared on different aphid hosts, such as *Aphis gossypii* Glover (Hemiptera: Aphididae) and *M. persicae,* where an innate preference for *A. gossypii* was found. Though it is currently unclear whether they studied *A. colemani* per se, as the identity of this species is not clear yet, it is possible that they have also studied *A. platensis,* as *A. colemani* is now a complex of cryptic, sibling species [49,75,76]. It was considered as a group, together with two other close species: *A. transcaspicus* and *A. platensis*, probably used as synonyms in several taxonomic descriptions [52,55,77]. Host fidelity is maximized in constant environments, where a continuous supply of aphid hosts is found [37]; e.g., parasitoids from the aphid hosts *S. avenae, Acyrthosiphon pisum* Harris (Hemiptera: Aphididae) alfalfa race and *A. pisum* pea race showed a preference for their natal host [51]. However, in their natural habitat, *R. padi* are not available throughout the cereal season, they arrive early in the season and by the end of the season, their populations decrease when *S. avenae* populations increase [78]. Therefore, a locally adapted *A. platensis* to *R. padi* could represent an evolutionary disadvantage. In this context, phenotypic plasticity allows *A. platensis* to adapt to new and/or unpredictable environmental conditions, maximizing their fitness [79,80,81].

Other reasons that explain the preference for one specific host, could be a better suitability of one host species, or that one aphid species has fewer defensive behaviours, meaning a shorter manipulation time. After an encounter, once a host is recognized through antennal evaluation, as in the case of all parasitoid species, *A. platensis* females perform different attack behaviours, such as abdomen bending and stings, which could result in a successful oviposition. However, after a host encounter, aphid parasitoids must overcome a variety of aphid defensive behaviours in response to their attacks, which could have an effect on their oviposition behaviour, leading to host rejection [59,72]. In this sense, *M. persicae* exhibited more defensive behaviours than *R. padi* (two of the three evaluated). Indeed *M. persicae* is well known for jerking with its abdomen, as well as walking away (escaping) and kicking [54]. Interestingly, female parasitoids originated from *M. persicae* elicit a lower response to *R. padi,* which kicked less, when compared to female parasitoids originated from *R. padi* which elicit a greater defensive response in *R. padi.* Apparently, *M. persicae* is a more difficult host to deal with, females preferred *R. padi* (which kick less) over *M. persicae,* even though they spent a similar amount of time handling the two aphid species. In contrast, *R. padi* kicked less and spent less time escaping, but the proportion of encounters with cornicular secretions was higher. This latter behaviour is common in *R. padi* compared to other aphid species [82], as it contains an alarm pheromone (β-farnesene), resulting in a deterrent effect on different parts of the parasitoid body [59]. However, in the case of *A. platensis,* this does not seem to affect its ability to use *R. padi* as a host. Moreover, as discussed in Ortiz et al. [83], β-farnesene could be an important cue for host detection in some parasitoids. Further research in the field should aid in the understanding of its role for host recognition and patch exploitation. In addition, β-farnesene can be used to alert other nearby aphid individuals to prevent damage [59], although this was not evaluated in this study, this should be taken into account for further investigations. In order to cope with aphid defences, parasitoids have evolved different behavioural and physiological mechanisms to efficiently deal with them, from changes in the host exploitation strategies to changes in the host oviposition [84,85]. For instance, according to our results, the handling time ending in a successful oviposition, was shorter in females coming from *R. padi*, which was the preferred host. In general, shorter handling times increase the reproductive success of a female parasitoid, which has a limited time available to locate suitable hosts, thereby reducing its vulnerability and mortality [86,87]. To decrease handling time, parasitoids may avoid hosts that are potentially difficult to handle, which is an adaptive behaviour [88]. The latter could help us understand why females of *A. platensis* avoided handling *M. persicae* (which showed more defensive behaviours) over *R. padi* (easier to handle). However, this could be related to the time spent wing fluttering. It was observed that different species of Aphidiinae spend different lengths of time wing fluttering (higher or lesser) when attacking a host, and it was shown, that when it occurs, less aphid defensive behaviours are produced, especially cornicular secretions [56]. Our results showed that females originating from *R. padi* spent less time fluttering their wings, however, irrespective of origin, females spent more time wing fluttering on *R. padi* than on *M. persicae.* Likewise, regardless of origin, the proportion of encounters with cornicular secretions was higher on *R. padi* than on *M. persicae*. Therefore, a different pattern among wing fluttering and cornicular secretion in *R. padi* was found in this study. However, although *R. padi* produced higher cornicular secretions, they were still preferred by *A. platensis* females.

As *A. platensis* populations were collected and reared on both hosts, they were expected to survive and develop on both aphid hosts. Here, in non-choice situations, *A. platensis* females were able to oviposit on *M. persicae* with no difference in the parasitism rates and the development time, however, in our study, regardless of the origin of the female, the progeny was always heavier when developing on *R. padi* (Figure 8B). This was irrespective of whether we offered aphids of the same initial size to females of *A. platensis* in order to provide the same amount of resources (second instar of *R. padi* and third instar of *M. persicae*). As host size is an important feature in parasitoid fitness [31,70,71] it has a positive relationship with the fitness of parasitoid progeny, as larger hosts usually contain more resources than smaller hosts [29,30,40,72,89,90].Thus, host size determines the host quality in which the progeny will develop [70]. Additionally, as proposed by Sequeira and Mackauer [91], a suitable host should be susceptible to parasitism and provide at least the minimum nutritional and physiological requirements for parasitoid development. Therefore, nutritional levels are important features (i.e., amino acid and lipid content) in the aphid hosts, which would explain their better physiological suitability. Due to the nutritional value of aphids (i.e., fatty acids and calories), these contents could have consequences at the third trophic level [92], mainly for parasitoid larvae development and later in reproduction [92,93].

In general, low parasitism was observed on both aphid hosts under laboratory conditions (Figure 6A), however, the emergence rate was higher on *R. padi* from females of both origins. These results suggest that *R. padi* would be a slightly more profitable host, however, *M. persicae* is also suitable. This is in contrast with the results of, Ode et al. [87] that measured the suitability of four aphid species for the closely related species parasitoid *A. colemani*: *A. gossypii*, *M. persicae*, *R. padi*, and *Schizaphis graminum* (Rondani) (Hemiptera: Aphididae). They showed that *R. padi* is a less suitable and a poor-quality host, compared to the other three Israelian aphid species tested, yet it appears to be a more favourable host, due to the larger size of parasitoids developing in it. Therefore, a trade-off between larval survival and body size among *R. padi* and *A. gossypii* is observed. The higher profitability of *R. padi* can explain that this species is the preferred host by *A. platensis*. However, our study also shows that *M. persicae* can easily be acceptable as there is no host fidelity, especially if *R. padi* populations are not present or are a scarce resource.

Given the results of preference and development obtained in this laboratory study, it could help explain the biological control of orchard pest results in the field when using cereal cover crops for alternative hosts such as *R. padi*. Therefore, we could expect that at the beginning of the season (in winter), *A. platensis* would attack *R. padi* in cereals (alternative host) as *M. persicae* is not present in orchards. Therefore, when the first populations of *M. persicae* on *Prunus* trees would appear and start damaging trees (early spring), there would be a short overlap of both crops (cereals and *Prunus* trees). However, in early spring, populations of *R. padi* would leave the cereals and move to other gramineous habitats, as they cannot colonize *Prunus domestica* (Linnaeus) and *P. persica*. Hence, as both hosts are suitable for the development of *A. platensis* (performance assay), when *R. padi* populations are no longer available, we can expect that the parasitoids could change hosts to *M. persicae* in *Prunus*, controlling their populations and improving biological control. However, since host selection of the most profitable aphid host by a female parasitoid includes a variety of other interacting factors as chemical and visual cues on field, further studies on host finding, searching behaviour are important for understanding the mechanisms involved in this more complex system.

## 5. Conclusions

From our results, we were able to obtain some evidence of the host foraging behaviour of *A. platensis* females, when faced with two different aphid species, as well as information regarding the interactions between them. This could have implications for conservation biological control, by ensuring that the alternative hosts used, provided the expected positive effects on beneficial organisms to control pests. By performing thorough laboratory experiments, we could obtain a first approach with the aim of establishing more efficient pest management strategies in orchard systems.

## Figures and Tables

**Table 1:** Choice experiment. Generalized estimating equations (GEE) showing the effect of the origin host and chosen aphid and the interaction between these two factors on the different behaviours of *A. platensis* and of the aphids *R. Padi* and *M. Persicae*. For each level, the degrees of freedom (df), the Chi-square statistical test and the *p*-value are represented.

Variables	Origin			Chosen			Interaction		
	df	*X* ^2^	*p*-Value	df	*X* ^2^	*p*-value	df	*X* ^2^	*p*-Value
First aphid perceived	1	1.08	0.30	-			-		
Ovipositor probing	1	0.25	0.62	1	0.65	0.42	1	3.05	0.08
Host acceptance	1	14.50	*1.4 × 10^−4^*	-			-		
Handling time for oviposition	1	11.62	*6.5 × 10^−4^*	1	1.67	0.19	1	0.06	0.81
Wing fluttering	1	13.70	*2.2 × 10^−4^*	1	6.00	*0.01*	1	0.00	0.98
Aphid kicking	1	1.79	0.18	1	10.82	0.37	1	3.82	*0.05*
Aphid escaping	1	2.04	0.15	1	20.97	*4.7 × 10^−6^*	1	0.61	0.44
Cornicular secretions	1	0.00	0.97	1	4.51	*0.03*	1	0.03	0.87

**Table 2:** Profitability assay. Generalized linear models (GLM) and Generalized linear mixed models (GLMM) showing the effect of the origin host, tested aphid species (*M. Persicae* and *R. Padi*) and the interaction between these two factors on the profitability traits of *A. platensis*. For each level, the degrees of freedom (df), the Chi square statistical test and the *p*-value are represented.

Variables	Origin			Tested			Interaction		
	df	*X* ^2^	*p*-Value	df	*X* ^2^	*p*-Value	df	*X* ^2^	*p*-Value
Parasitism rate ^1^	1	0.04	0.84	1	2.54	0.11	1	0.00	0.95
Emergence ^2^	1	1.01	0.32	1	4.86	*0.03*	1	0.51	0.47
Total developmental time ^2^	1	0.02	0.88	1	0.59	0.44	1	1.13	0.29
Tibia length ^1^	1	0.00	0.99	1	1.56	0.21	1	0.04	0.84
Fresh body mass ^1^	1	2.18	0.14	1	7.43	*0.01*	1	0.08	0.78

^1^ Generalized linear models (GLM), ^2^ Generalized linear mixed models (GLMM).
